# Incidence and determinants of hematotoxicity in acute lymphoblastic leukemia children who received 6-mercaptopurine based maintenance therapy in Addis Ababa, Ethiopia

**DOI:** 10.1371/journal.pone.0286544

**Published:** 2023-06-02

**Authors:** Awol Mekonnen Ali, Haileyesus Adam, Daniel Hailu, Marieke J. H. Coenen, Rawleigh Howe, Teferra Abula

**Affiliations:** 1 Department of Pharmacology and Clinical Pharmacy, School of Pharmacy, College of Health Sciences, Addis Ababa University, Addis Ababa, Ethiopia; 2 Department of Pediatrics and Child Health, School of Medicine, College of Health Sciences, Addis Ababa University, Addis Ababa, Ethiopia; 3 Department of Human Genetics, Radboud Institute for Health Sciences, Radboud University Medical Center, Nijmegen, The Netherlands; 4 Armauer Hansen Research Institute, Addis Ababa, Ethiopia; Kaohsuing Medical University Hospital, TAIWAN

## Abstract

**Introduction:**

The maintenance phase of acute lymphoblastic leukemia treatment is the final and longest stage of treatment, mainly focused on antimetabolite therapy. This phase is essential to eliminate residual leukemic clones and prevent relapse. However, dose-limiting hematotoxicity is a major problem during this phase resulting in dose reduction or treatment discontinuation.

**Objective:**

In this cohort study, the clinical features and risk factors of hematological toxicity during the maintenance phase of treatment were analyzed in pediatric patients from Ethiopia.

**Methods:**

A total of 160 patients from Tikur Anbessa specialized hospital were included in the study of which 142 had sufficient data available for analysis. Patient characteristics as well as information about the care-givers, sides-effects as reported by the care-givers and clinical factors were collected. Bivariable followed by multivariable analysis was performed to investigate which factors were associated with hematological toxicity during the maintenance phase.

**Results:**

During the first six months of maintenance phase treatment grade 4 neutropenia was detected in 52.8% of the patients. The risk of developing grade 4 neutropenia was increased by about two fold in children with the age of 6 years and less compared to those with the age of more than 6 years. Similarly, the rate of developing grade 4 neutropenia among children with less than 4,500 maintenance day 1 white blood cell counts was significantly higher than that of children with normal maintenance day 1 white blood cell counts (AHR 2.477, 95% CI **=** 1.461–4.200, *p* = 0.001).

**Conclusion:**

In conclusion, child’s age and day 1 maintenance white blood cell/absolute neutrophil counts significantly affected the occurrence of grade 4 hematotoxicity. Close monitoring for white blood cell and absolute neutrophil counts during maintenance phase treatment is recommended for early diagnosis of hematotoxicity.

## Introduction

Acute lymphoblastic leukemia (ALL) is the most common childhood cancer. It is estimated to account for 19% of total childhood cancer incidences globally [1]. In Ethiopia, the annual incidence of childhood cancers has been estimated between 3,707 and 6000 cases, with leukemia being the common (29%), followed by non-Hodgkin’s lymphoma, Wilms tumor, and retinoblastoma [2, 3]. Acute leukemia is observed in 89% of all leukemia cases in children in Ethiopia, ALL accounts for 91% of these cases and 9% can be attributed to acute myeloid leukemia [2]. The outcomes for acute leukemia are lower in low- and middle-income countries (LMICs) compared to developed ones [4]. Lack of resources for both the patients and healthcare professionals is the key factor, leading to delayed diagnosis and/or adverse clinical outcomes [5]. Socioeconomic factors like malnutrition, high infection rate, comorbidities, and disease biology also contribute to the inferior survival observed in these settings [4, 5]. The cure rates range from 20% to 70% in LMICs compared to >80% in high income countries [5, 6].

The disease risk group, and thus treatment intensity, is mainly determined by disease stage in LMICs. Hence, patients are stratified into three disease risk groups based on physical examination, age, initial white blood cell count (WBC), central nervous system (CNS) status, and early response [7]. To optimize treatment chemotherapies are combined differently in each phase of treatment based on the disease risk groups [8]. To maintain long-term remission, ALL patients require maintenance phase therapy for up to 2.5 years from the start of treatment with daily oral 6-mercaptopurine (6-MP), weekly oral methotrexate (MTX), and monthly vincristine/steroid pulses [7]. Maintenance phase therapy is directed to keep patients in remission [9], however, it can lead to hematotoxicity (including anemia, leukopenia, neutropenia, and thrombocytopenia) [10, 11]. Hematotoxicity is the main dose-limiting toxicity of chemotherapy [12]. ALL patients with severe neutropenia or thrombocytopenia may require 6-MP dose reduction or even discontinuation [10]. In addition, severe neutropenia can lead to mortality as a result of vulnerability to infections and sepsis [13]. During maintenance phase therapy toxicity is a vital issue to consider; it is the main reason for drug discontinuation, leading to relapse. Moreover, hematotoxicity can be life-threatening, and hence knowledge on factors that influence hematotoxicity can be of added value for the treatment of the patients.

In contrast to low and middle income countries the incidence and factors associated with chemotherapy induced hematotoxicity in ALL are well investigated in developed countries. Therefore, this study aimed to determine the incidence and predictors of hematotoxicity during 6-mercaptopurine based maintenance therapy among pediatric ALL patients from Ethiopia.

## Methods

### Study setting and patient recruitment

This cohort study was carried out at Tikur Anbessa specialized hospital (TASH), a tertiary care center affiliated with College of Health Sciences, Addis Ababa University, Addis Ababa, Ethiopia. A total of 160 pediatric ALL patients were enrolled from 2019 to 2021 at the pediatric oncology department of TASH. TASH is the only compressive cancer care and treatment center for both children and adults in Ethiopia. The pediatric oncology department of the hospital is located on the 7D, seventh floor of the main building. The outpatient pediatric oncology department is located in the first floor of cancer clinic of the hospital. Patients with renal disease, liver disease, and heart failure were excluded from the study. Patients were stratified into standard risk (SR), intermediate risk (IR), and high risk group (HR) based on a physical examination, age, initial white blood cell count, central nervous system status, and early prednisolone response. Written informed consent was obtained from all participants’ caregivers before study enrolment. Ethical approval to conduct this study was obtained from the Institutional Review Board of the College of Health Sciences, Addis Ababa University, the Armauer Hansen Research Institute Ethical Review Committee, and the Ethiopian National Research Ethics Review Committee. The identities of the study participants were kept confidential. Patients were treated using a protocol for low- and middle-income countries [7]. The maintenance phase is always initiated with 75 mg/m^2^ of 6-MP based on the protocol for North America. The dose of 6-MP is either discontinued or reduced primarily due to severe neutropenia. Furthermore, trimethoprim/sulfamethoxazole (TMP/SMX) at a dose of TMP 5 mg/kg/d 3 times per week was given for *Pneumocystis jirovecii* prophylaxis as a co-medication.

### Data collection

Patients’ demographic, clinical characteristics including clinical presentation, complete blood count (CBC), peripheral morphology, peripheral and bone marrow blast, and risk group were collected from medical records. Clinical profiles such as CBC, fever, emergency admission, dose reduction, and drug discontinuation were collected for the first 6-months after start of the maintenance phase. A CBC was performed at a 4-week interval unless it was indicated for any clinical reasons. Family demographics, economic status, child feeding, drug adherence, and reported adverse events were collected from caregivers using questionnaires.

Medication adherence was assessed using the four-item Morisky, Green, and Levine Medication Adherence Questionnaire (MGL). The adherence scores were calculated, and participants were categorized as low adherence (3 or 4 items answered Yes), moderate adherence (1 or 2 item/s answered Yes), and high adherence (0 item answered Yes) [14].

Weight-for-height z-scores (WHZ), height-for-age z-scores (HAZ), weight-for-age z-scores (WAZ), and Body mass index (BMI) for age Z score (BAZ) were computed using WHO Anthro Version 3.2.2 for children up to five years. The WAZ, HAZ, and BAZ were computed using WHO AnthroPlus Version 1.0.4 for children older than five years. The WAZ was generated for children younger than 10 years. Children with z-scores of less than −2 standard deviations (SDs) for HAZ, WHZ, WAZ, and BAZ were classified as stunted, wasted, underweight, and thin, respectively [15].

### Study outcomes

Grading of hematologic toxicity was based on the Common Terminology Criteria for Adverse Events (CTCAE), version 4.0 [16]. Accordingly, toxicity classified for grade 4 when WBC <1000/mm^3^; ANC <500/mm^3^; anemia <6.5 g/dl and thrombocytopenia <25,000/mm^3^. The primary outcome measure was the occurrence of grade 4 neutropenia during maintenance treatment. The secondary outcomes were the drug discontinuation, neutropenic fever and early-onset grade 4 leukopenia/neutropenia. Early-onset leukopenia/neutropenia was defined as the occurrence of leukopenia/neutropenia during the first 60 days of the maintenance therapy [17].

### Statistical analysis

Data were analyzed using SPSS version 26. Study participants’ demographic characteristics, anthropometric, and clinical profiles were presented using descriptive statistics as median (IQR) or as frequency and percentages.

Risk factor analysis and hazard ratios for the primary and secondary outcomes were calculated using Cox regression. Bivariable followed by multivariable analysis were performed to identify predictive factors associated with hematotoxicity. In all multivariable analysis, variables with *p* ≤ 0.20 in bivariable analysis were used with enter as variable selection method. To avoid the interaction between WBC and ANC in the regression, two multivariable regression models were used. First, all variables selected from bivariable analysis, except ANC, were entered into multivariable model. In model 2, all variables except WBC were modeled. Significance threshold was set at p-values <0.05. Variable used in the association study for the primary outcome are:—child’s age, sex, place of residence, caregivers’ educational level, marital status, adherence, HAZ, BAZ, food item, risk group, WBC and ANC.

## Results

### Socio-demographic characteristics

In total 160 children participated in the study, eighteen children were not included in statistical analysis due to incomplete data. Socio-demographic characteristics of participants included in the statistical analysis are presented in Table 1. 92 (64.8%) were boys and 50 (35.2%) were girls. The mean age was 6.2±3.1 years with 55.6% under the age of 6. Most study participants were from urban area. A majority of the caregivers were married (89.4%) and 22.5% of caregivers had no schooling. 45.1% of the children came from families with more than five members and the majority of the caregivers (90.2%) were biological parents of the children.

**Table 1:** Socio-demographic characteristics of the study population in outpatient pediatric oncology department of TASH (n = 142).

		Number (percentage)
Child’s sex	Male	92 (64.8%)
	Female	50 (35.2%)
Child’s age (years)	≤ 6	79 (55.6%)
	> 6	63 (44.4%)
Caregivers’ age (years)	18–25	14 (9.9%)
	≥ 26	128 (90.1%)
Caregivers’ sex	Male	72 (50.7%)
	Female	70 (49.3%)
Caregivers’ marital status	Single	7 (4.9%)
	Married	127 (89.4%)
	Divorced	3 (2.1%)
	Widowed	5 (3.5%)
Place of residence	Urban	82 (57.7%)
	Rural	60 (42.3%)
Caregivers’ educational level	Can’t read or write	32 (22.5%)
	Elementary	32 (22.5%)
	Secondary education	48 (33.8%)
	Vocational education	9 (6.3%)
	University education	21 (14.8%)
Caregivers’ occupation	House wife	44 (31.0%)
	Farmer	34 (23.9%)
	Daily Laborer	4 (2.8%)
	Government Employee	19 (13.4%)
	Retired	1 (0.7%)
	Merchant/Trade	29 (20.4%)
	Self employed	11 (7.7%)
Caregivers’ relation to child	Mother	62 (43.7%)
	Father	66 (46.5%)
	Sister	3 (2.1%)
	Brother	4 (2.8%)
	Aunt	2 (1.4%)
	Uncle	2 (1.4%)
	Grand mother	3 (2.1%)
Family size	≤ 5	78 (54.9%)
	> 5	64 (45.1%)
Average monthly income of the family (ETB)	Low (446–1200)	43 (30.3%)
	Average (1201–2500)	26 (18.3%)
	Above average (2501–3500)	29 (20.4%)
	High (>3501)	44 (31.0%)

All participants were in remission during maintenance phase of treatment. A bit more than half (53.5%) of the children obtained food containing protein and 92.3% used three or more meals daily. Based on the WHO Z-score definition (<-2 ZS), 14.3% of the children were wasted, 22.1% were underweight, 24.6% were stunted, and 21.8% were thin. The medication adherence scores revealed that 78.2% of children have a high level of adherence, while 21.8% have a medium level of adherence as depicted in Table 2. Among medium adherents, 48.4% forgot to give medicines regularly, 22.6% were careless about giving medications, and 29% stopped medication on feeling worse.

**Table 2:** Frequency distribution of child general heath, nutritional status, and adherence in outpatient pediatric oncology department of TASH (n = 142).

		Number (percentage)
Child general health	Excellent	3 (2.1%)
	Very good	97 (68.3%)
	Good	42 (29.6%)
WHZ	Wasted	8 (14.3%)
	Normal	48 (85.7%)
WAZ	Underweight	25 (22.1%)
	Normal	88 (77.9%)
HAZ	Stunted	35 (24.6%)
	Normal	107 (75.4%)
BAZ	Thin	31 (21.8%)
	Normal	111 (78.2%)
Number of meals per day	2	11 (7.7%)
	3	68 (47.9%)
	4	62 (43.7%)
	5	1 (0.7%)
Food item	Meat, egg, Milk, Vegetable	76 (53.5%)
	Other	66 (46.5%)
Adherence	High	111 (78.2%)
	Medium	31 (21.8%)

WHZ = Weight-for-height z-scores, HAZ = Height-for-age z-scores, WAZ = Weight-for-age z-scores, BAZ = Body mass index (BMI) for age Z score

The patient baseline clinical profiles are depicted in S1 Table in S1 File. Hepatomegaly (60.4%) and splenomegaly (54.7%) presented in isolation or in combination. A bit more than half of the patients (51.4%) were classified as high risk, all others fall in the standard risk group. BSA of the children ranged from 0.46 to 1.53 m^2^ with a median value of 0.79. The median WBC counts at diagnosis was 12,340/mm^3^. One-fourth of the patients (23.4%) had WBC counts over 50,000/mm^3^. The median WBC and ANC were 3,500/mm^3^ and 1,600/mm^3^ respectively at the commencement of the maintenance phase of treatment.

### Caregivers’ perceptions of chemotherapy-related side effects

**Fig 1:**
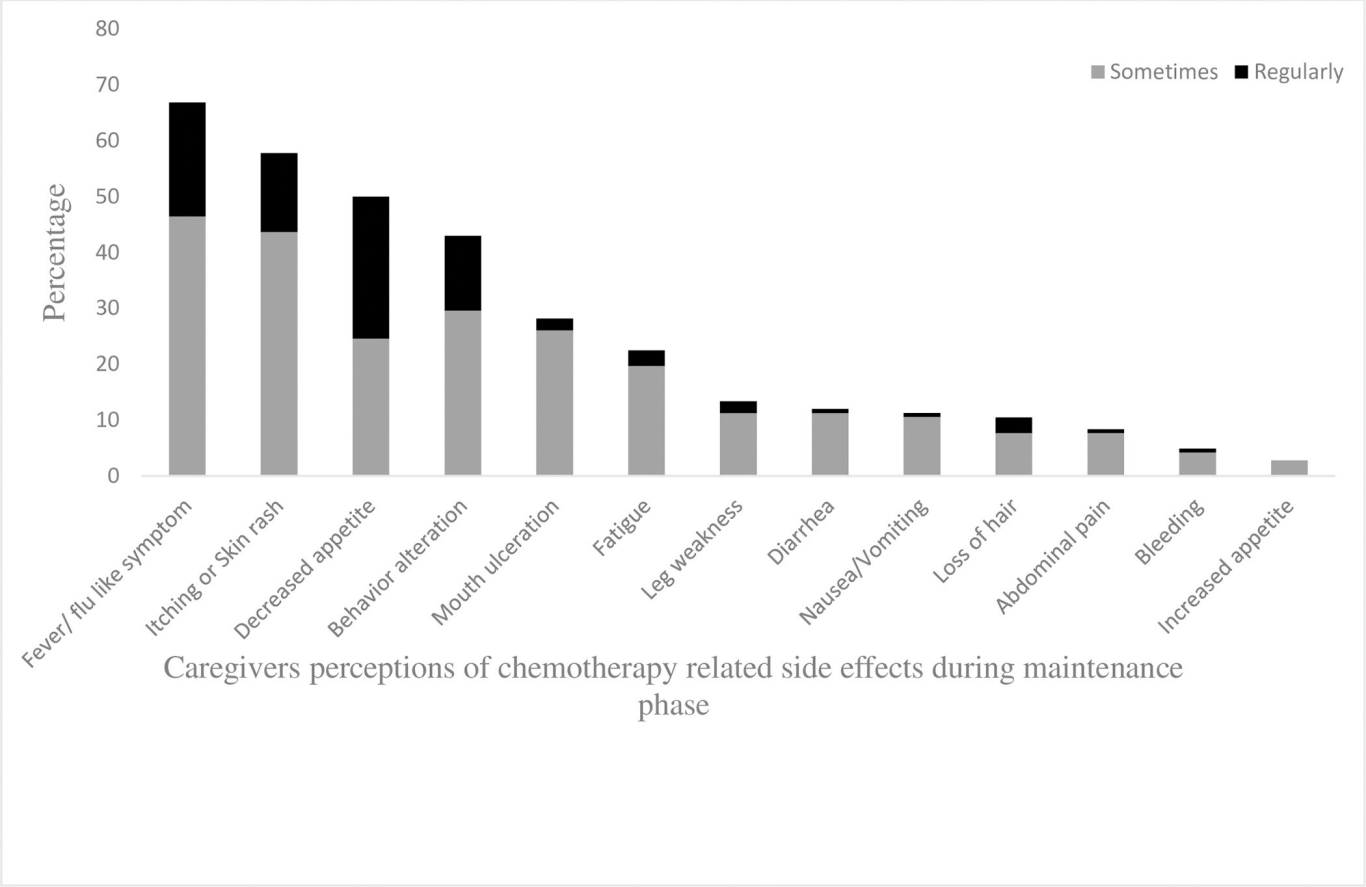
Perceptions of caregivers of children toward the frequency and the severity of chemotherapy related side effects during maintenance phase (n = 142).

Fig 1 shows the frequency of caregivers’ perceptions of chemotherapy-related side effects. Fever/flu-like symptoms were the most frequently noted side effect with 66.9% of the caregivers reporting this. Itching or skin rash (57.8%), decreased appetite (50%), and behavior alterations (43%) were consecutively the second, third, and fourth most frequent side effects.

### Incidence of grade 4 hematotoxicity, treatment interruption, and emergency admission

A description of grade 4 hematotoxicity, and treatment interruption during the first 6 months of maintenance treatment is presented in Table 3. Early-onset leukopenia was observed in 19.7% of the study participants. About one-third (32.4%) of patients developed early-onset neutropenia. During the first six months of maintenance phase treatment, leukopenia and neutropenia were seen in 31.7% and 52.8% of the patients, respectively. A bit more than half (58.5%) of the patients required drug interruption. Sixty-five (45.8%) patients were admitted to the emergency unit mainly due to neutropenic fever (31%). Anemia was reported in 13.4% of the patients. Only 5.6% experienced thrombocytopenia.

**Table 3:** Incidence of grade 4 hematotoxicity, treatment interruption, and emergency admission, among the study participants (n = 142) during the first 6 months of maintenance therapy in outpatient pediatric oncology department of TASH.

	Number (percentage)
Early-onset leukopenia	28 (19.7%)
Leukopenia	45 (31.7%)
Early-onset neutropenia	46 (32.4%)
Neutropenia	75 (52.8%)
Treatment interruption	83 (58.5%)
Neutropenic fever	44 (31%)
Emergency admission	65 (45.8%)
Anemia	19 (13.4%)
Thrombocytopenia	8 (5.6%)

6-MP = 6-Mercaptourine

### Predictors of grade 4 neutropenia

Table 4 provides an overview of the risk factors linked to the development of chemotherapy-induced grade 4 neutropenia in the bivariable (only factors with a p<0.02 are shown) and multivariable cox proportional hazard regression analysis. Bivariable cox proportional hazard regression analysis showed that the child’s age, and day 1 maintenance WBC were significantly associated with grade 4 neutropenia. Multivariable analysis revealed that the rate of developing grade 4 neutropenia was about 2 times higher in a child under the age of 6 compared to those above 6 (model 1 and 2). The rate of developing grade 4 neutropenia among children with a WBC less than 4,500 (at day 1 of the maintenance therapy) was 2.477 times (AHR 2.477, 95% CI = 1.461–4.200, *p* = 0.001) higher than that of children with normal WBC counts (model 1). Similarly, the risk of developing grade 4 neutropenia among children with ANC less than 2,500 (at day 1 of the maintenance therapy) was 2.11 times (AHR 2.11, 95% CI = 1.105–4.029, *p* = 0.024) higher than that of children with normal ANC counts (model 2).

**Table 4:** Cox proportional hazard regression results for incidence of grade 4 neutropenia in outpatient pediatric oncology department of TASH (n = 142).

Predictor Factor	Multivariable					
	Bivariable		Model 1		Model 2	
	CHR (95% CI)	*p*-value	AHR (95% CI)	*p*-value	AHR (95% CI)	*p*-value
CAY						
> 6	1		1		1	
≤ 6	1.891 (1.174–3.045)	0.009	2.189 (1.351–3.548)	0.001	2.09 (1.291–3.383)	0.003
MD1WBC						
≥4500	1		1			
<4500	2.155 (1.28–3.63)	0.004	2.477 (1.461–4.200)	0.001		
MD1ANC						
≥4500	1				1	
<4500	1.806 (0.952–3.426)	0.07			2.11 (1.105–4.029)	0.024

CAY = Child’s age (Years), MD1WBC = Maintenance day 1 WBC, MD1ANC = Maintenance day 1 ANC, AHR = Adjusted hazard ratio, CHR = Crude hazard ratio

### Predictors of grade 4 early-onset leukopenia/neutropenia, treatment interruption and neutropenic fever

Child’s age, and day 1 maintenance WBC counts showed association with a treatment interruption during bivariable regression analysis (Table 5). Child’s age (≤ 6 year) (AHR 2.1, 95% CI = 1.33–3.317, *p* = 0.001), and day 1 maintenance WBC counts (AHR 2.024, 95% CI = 1.249–3.282, *p* = 0.004) were found to be independently associated with a treatment interruption after multivariable regression analysis (model 1). In model 2, low ANC count (AHR 1.938, 95% CI = 1.065–3.527, *p =* 0.03), and child’s age (≤ 6 year) (AHR 2.018, 95% CI = 1.282–3.179, *p* = 0.002) were independent risk factors of treatment interruption. Only child’s age contributed significantly to the occurrence of neutropenic fever in the bivariable analysis (Table 5). Child’s age, and day 1 maintenance WBC counts were significantly associated with early-onset grade 4 leukopenia/neutropenia following bivariable regression analysis (S2 Table in S1 File). Multivariable analysis showed that child’s age (≤ 6 year) (AHR 3.024, 95% CI = 1.282–7.136, *p* = 0.012), and day 1 maintenance WBC counts (≤ 4500) (AHR 4.498, 95% CI = 1.555–13.01, *p* = 0.006) were independent predictors of early-onset leukopenia. Similarly, child’s age (≤ 6 year) (AHR 2.919, 95% CI = 1.505–5.659, *p =* 0.002), and day 1 maintenance WBC counts (≤ 4500) (AHR 2.73, 95% CI = 1.349–5.524, *p* = 0.005) were found to be independent risk factor for early-onset neutropenia (S2 Table in S1 File, model 1).

**Table 5:** Cox regression results for neutropenic fever and treatment interruption among the study participants in outpatient pediatric oncology department of TASH (n = 142).

SNPs	Neutropenic fever Treatment interruption							
	Bivariable		Bivariable		Model 1 (Multivariable)		Model 2 (Multivariable)	
	CHR (95% CI)	*p*-value	CHR (95% CI)	*p*-value	AHR (95% CI)	*p*-value	AHR (95% CI)	*p*-value
CAY								
> 6	1		1		1		1	
≤ 6	2.448 (1.26–4.754)	0.008	1.848 (1.179–2.896)	0.007	2.1 (1.33–3.317)	0.001	2.018 (1.282–3.179)	0.002
MD1WBC								
≥4500	1		1		1			
<4500	1.241 (0.658–2.34)	0.505	1.753 (1.09–2.82)	0.021	2.024 (1.249–3.282)	0.004		
MD1ANC								
≥4500	1		1				1	
<4500	1.03 (0.495–2.142)	0.93	1.678 (0.928–3.034)	0.087			1.938 (1.065–3.527)	0.03

CAY = Child’s age (Years), MD1WBC = Maintenance day 1 WBC, MD1ANC = Maintenance day 1 ANC, AHR = Adjusted hazard ratio

## Discussion

In this study we investigated incidence of grade 4 hematotoxicity and factors affecting maintenance treatment of patients with ALL. This study shows a higher incidence of grade 4 neutropenia (52%) as compared to previous reports performed in high income countries [18, 19]. A relatively close incidence of grade 4 neutropenia (47%) was reported in studies conducted in China [20] and Thailand [21]. Conversely, a higher incidence of grade 4 neutropenia was reported in a study performed in Korea [22] and Sweden [23]. This study also showed that the incidence of 6-MP interruption and neutropenic fever were higher than that of the previous reports [24, 25]. However, other studies reported higher treatment interruption than the current study [18, 26]. In general, there are differences in the distribution of 6-MP induced toxicity across the globe, and this discrepancy can be attributed to several factors, including patient characteristics, follow-up period, genetic variation, and differences in 6-MP dose and dose adjustment protocol.

This is one of the very few studies that shows data of side-effects of maintenance therapy as reported by the care-givers. This study revealed that fever/flu-like symptoms followed by itching or skin rash, decreased appetite, and behavior alterations were the most frequently noted side effect. Another study from Indonesia [27] however, reported that behavior alterations were the most frequently noted side effect followed by increased appetite and infection. Higher number of fever/flu-like symptoms in the current study might be due to neutropenia.

Child’s age and day 1 maintenance blood counts significantly influence the occurrence of neutropenia. Risk factors for chemotherapy induced hematotoxicity can be categorized into three classes: disease factors; patient characteristics (age, comorbidity, abnormal laboratory results before therapy, and nutrition); and therapeutic factors (types and doses of chemotherapy) [10, 28]. This is among a few studies that assessed factors associated with 6-MP-based chemotherapy side effects in the maintenance phase of pediatric ALL treatment. Despite the importance of neutropenia as the primary dose-limiting toxicity of chemotherapy [29], its risk factors have not been well investigated in pediatric ALL patients, particularly in Ethiopia. Overall the result of this study revealed that child’s age, and day 1 maintenance WBC/ANC counts were independent predictors of grade 4 neutropenia. Patients who developed grade 4 neutropenia had a significantly lower age and day 1 maintenance WBC/ANC counts than patients who did not develop grade 4 neutropenia. These findings are in agreement with previous reports on chemotherapy induced neutropenia in adult populations, who documented younger age [12] and low baseline WBC/ANC counts [30] as significant risk factors. Pervious study in pediatric ALL patients also showed that younger age was associated with an increased risk of neutropenia [23].

Failure of neutrophil production in the bone marrow or peripheral neutrophil destruction may cause neutropenia. There are multiple acquired causes of neutropenia such as infection, nutritional deficiencies, copper deficiency, protein malnutrition, immune reactions, and chemotherapy-induced neutropenia [10, 31]. However, the findings of this study depicted that patients’ gender, nutritional status, and risk group were not related to neutropenia. These observations are consistent with the study by Rosdiana *et al*., [10], where patients’ gender, BMI, nutritional status, and risk group were not associated with neutropenia in pediatric ALL patients. Another study also showed that patients’ gender, BMI, and disease risk stratification and stage were not related to the neutropenia occurrence in pediatric cancer patients [32]. However, these findings contradict several reports in adult populations, where BMI, disease stage, and gender are significant predicting factors for the occurrence of neutropenia [29, 33, 34]. In addition to patients’ characteristics, no significant association between any of the caregivers’ characteristics (age, gender, place of residence, educational level, and marital status) and the risk of neutropenia were identified in this study.

This study highlighted that younger age is a risk factor for febrile neutropenia. This observation is in line with pervious study which indicates that younger age is a risk factor for febrile neutropenia [23]. However, another study showed that age was not a risk factor for febrile neutropenia [35]. Neutropenia is the main reason for treatment discontinuation and dose reduction in this cohort of patients. Age and day 1 maintenance WBC/ANC counts were shown to be associated with the treatment interruption. Patients with a younger age (*≤*6 years) and low day 1 maintenance WBC/ANC counts had more often 6-MP interruption compared to a patient with older age and normal day 1 maintenance WBC/ANC counts. It is widely accepted that treatment outcome is related to treatment intensity in many drug sensitive cancers, including childhood ALL [36]. The delivered dose intensity is a major determinant of the treatment outcome [37]. Thus, every effort should be made to reduce the occurrence of treatment interruption and our study suggests that a focus on younger children and a low WBC/ANC count might be worthwhile.

Despite the small sample size and a single institutional study, this study provides essential information on factors associated with hematologic toxicities. Unfortunately, studies reporting similar data are limited. Multi-centered future studies with a large sample size are required to further validate these findings. Close monitoring for WBC and ANC counts during maintenance phase treatment is recommended for early diagnosis of hematotoxicity.

## Conclusions

In conclusion, this study showed a high incidence of hematotoxicity particularly grades 4 neutropenia in ALL patients who underwent 6-MP treatment during the maintenance phase of treatment. Child’s age and day 1 maintenance blood counts significantly affect the occurrence grade 4 hematotoxicity. Patients with the age of 6 year and younger and low day 1 maintenance WBC/ANC counts need prior support before initiation of chemotherapy. However additional studies are necessary to confirm the findings of this study.

## Supporting information

S1 File (DOCX)

S1 Data (XLSX)
