# Supplementary material for: Incidence and determinants of hematotoxicity in acute lymphoblastic leukemia children who received 6-mercaptopurine based maintenance therapy in Addis Ababa, Ethiopia
Source: PLoS One. 2023 Jun 2;18(6):e0286544. doi: 10.1371/journal.pone.0286544 (PMC10237636; doi:10.1371/journal.pone.0286544)
Supplement: S1 File — (DOCX) [file pone.0286544.s001.docx]

Supplementary

Supplementary Table 1. Baseline clinical characteristics of study participants in outpatient pediatric oncology department of TASH (n=142).

|  | | Number (percentage) |
| --- | --- | --- |
| Hepatosplenomegaly | Hepatomegaly | 84 (60.4%) |
|  | Splenomegaly | 76 (54.7%) |
| RBC peripheral | Normocytic & normochromic | 84 (63.2%) |
|  | Anisopoikilocytosis | 12 (9%) |
|  | Difficult to comment | 37 (27.8%) |
| Peripheral morphology WBC | Normal | 21 (16.7%) |
|  | Increase | 65 (51.6%) |
|  | Decrease | 40 (31.7%) |
| Risk group | Standard risk | 69 (48.6%) |
|  | High risk | 73 (51.4%) |
| **Continuous variable** | | Median (IQR) |
| Body surface area (m^2^) (n=142) | | 0.79 (0.68-1.01) |
| WBC at diagnosis (cells/mm^3^) (n=137) | | 12340 (4500-44850) |
| Lymphocyte at diagnosis (%) (n=119) | | 77.7 (59.9-87.5) |
| ANC at diagnosis (cells /mm^3^) (n=126) | | 930 (351-3682) |
| Hemoglobin at diagnosis (gm/dL) (n=134) | | 7.55 (6.1-9.2) |
| Platelet at diagnosis (cells /mm^3^) (n=133) | | 35000 (18500-79000) |
| Peripheral blast (%) (n=122) | | 25 (6-54.25) |
| Bone marrow blast (%) (n=127) | | 88 (60-100) |
| WBC at the beginning of maintenance therapy (cells /mm^3^) (n=142) | | 3500 (2675-5450) |
| ANC at the beginning of maintenance therapy (cells /mm^3^) n=142) | | 1600 (1075-2352) |

**_ANC = Absolute neutrophil count, IQR = Interquartile range, RBC = Red blood cell WBC = White blood cell_**

| SNPs | Early-onset grade 4 leukopenia | | | | Early-onset grade 4 neutropenia | | | |
| --- | --- | --- | --- | --- | --- | --- | --- | --- |
|  | Bivariable | | Model 1 (Multivariable) | | Bivariable | | Model 1 (Multivariable) | |
|  | CHR (95% CI) | *p*-value | AHR (95% CI) | *p*-value | CHR (95% CI) | *p*-value | AHR (95% CI) | *p*-value |
| CAY  > 6  ≤ 6 | 1  2.603 (1.106-6.124) | 0.028 | 1  3.024 (1.282-7.136) | 0.012 | 1  2.594 (1.343-5.011) | 0.005 | 1  2.919 (1.505-5.659) | 0.002 |
| MD1WBC  ≥4500  <4500 | 1  3.934 (1.364-11.342) | 0.011 | 1  4.498 (1.555-13.01) | 0.006 | 1  2.38 (1.181-4.798) | 0.015 | 1  2.73 (1.349-5.524) | 0.005 |
| MD1ANC  ≥4500  <4500 | 1  1.377 (0.524-3.623) | 0.517 |  |  | 1  1.198 (0.578-2.482) | 0.628 |  |  |

Supplementary Table 2. Cox regression results for predictors of the early-onset grade 4 leukopenia and neutropenia in outpatient pediatric oncology department of TASH (n=142).

_CAY= Child’s age (Years), MD1WBC=Maintenance day 1 WBC, MD1ANC= Maintenance day 1 ANC, AHR = Adjusted hazard ratio_
